# Pediatric Sedation: A Global Challenge

**DOI:** 10.1155/2010/701257

**Published:** 2010-10-19

**Authors:** David Gozal, Keira P. Mason

**Affiliations:** ^1^The Sedation Service, Department of Anesthesiology and Critical Care Medicine, Hadassah University Hospital, Jerusalem 91120, Israel; ^2^Department of Anesthesia, Children's Hospital Boston, 300 Longwood Avenue, Boston, MA 02115, USA

## Abstract

Pediatric sedation is a challenge which spans all continents and has grown to encompass specialties outside of anesthesia, radiology and emergency medicine. All sedatives are not universally available and local and national regulations often limit the sedation practice to specific agents and those with specific credentials. Some specialties have established certification and credentials for sedation delivery whereas most have not. Some of the relevant sedation guidelines and recommendations of specialty organizations worldwide will be explored. The challenge facing sedation care providers moving forward in the 21st century will be to determine how to apply the local, regional and national guidelines to the individual sedation practices. A greater challenge, perhaps impossible, will be to determine whether the sedation community can come together worldwide to develop standards, guidelines and recommendations for safe sedation practice.

## 1. Introduction

Pediatric sedation is a challenge which spans all continents. Over the past decade, sedation has grown to encompass specialties outside of anesthesia, radiology, and emergency medicine. Until the 1990s, sedation in the United States was limited predominantly to delivery by anesthesiologists, radiologists, dental medicine, and emergency medicine physicians. It now encompasses other specialties which include gastroenterology, intensive care medicine, hospital medicine, pediatric medicine, and nursing [1–3]. Worldwide, however, the majority of pediatric sedation is still administered by anesthesiologists. All sedatives are not universally available and local and national regulations often limit the sedation practice to specific agents and those with specific credentials. Some specialties have established certification and credentials for sedation delivery whereas most have not [4–10]. The challenge is that there is no standardization of sedation practice, guidelines, and credentialing: Many specialties have guidelines and recommendations for their own practice, which may in fact contradict the guidelines set forth by other specialty societies [5, 11–13]. 

 The challenge facing sedation care providers moving forward in the 21st century will be to determine how to apply the local, regional, and national guidelines to the individual sedation practices. A greater challenge, perhaps impossible, will be to determine whether the sedation community can come together worldwide to develop standards, guidelines, and recommendations for safe sedation practice. Some of the relevant sedation guidelines and recommendations of specialty organizations worldwide will be explored. To our knowledge, this will be the first paper to present a comprehensive representation of guidelines across the specialties spanning the globe.

## 2. Models of Pediatric Sedation: A Global Tour

This paper will explore the existing sedation models, citing examples of sedation care delivered by different individual specialties. Each model and specialty have created their own set of guidelines and models for sedation administration. We have conducted a comprehensive review of the literature to present representative models of sedation delivery directed by different specialties. A summary of the representative models is presented in Table 1.

### 2.1. Anesthesiologist-Directed Sedation Model [14]

Most common in areas outside the United States, with the exception of countries which have limited anesthesia providers, is the delivery and oversight of sedation by anesthesiologists. The Hadassah University Hospital in Jerusalem was the first hospital in Israel to set up a Sedation Service. This Sedation Service is an example of an anesthesia-directed sedation program and was developed to involve a multispecialty team comprised of specially trained nurses, all with intensive care background, and pediatric anesthesiologists. All sedation is delivered by protocols which were developed by the Department of Anesthesia and approved by the Hospital.

**Figure 1:**
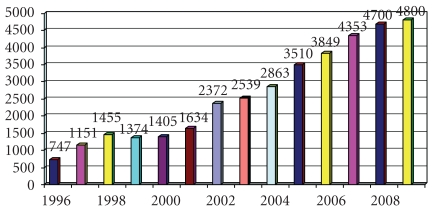
Sedation volume at Hadassah Hospital, Israel.

The Sedation Service provides an efficient framework for easing the pain and anxiety in a number of diagnostic or therapeutic procedures performed out of the operating room (OR). As the demand for procedural sedation has increased, so too has the sedation volume. Gradually, the sedation service has evolved to care not only for the pediatric population but also to provide sedation across the age spectrum to even include the elderly. The service has expanded to encompass sedation delivery to over 5000 patients a year, across all specialties in over 40 departments, institutes, and clinics within the Hospital (Figure 1). 

The sedation process begins before the patient arrives for the procedure. All patients are carefully screened for preexisting medical illness and appropriateness for sedation before arrival. For outpatients, a few days before the required procedure, a telephone evaluation is performed by the sedation nurse with the child's parents or guardian. For children in hospital, the physician caring for the child relays the pertinent clinical information and also provides the family with informational materials describing the sedation process. Much of the triage is done without the direct involvement of the anesthesiologist, following existing guidelines. Per protocol, however, anesthesiologists are consulted for patients who are American Society of Anesthesiologists (ASA) 3 and 4 [11]. Sedation is delivered in accordance with the American Academy of Pediatrics and American Society of Anesthesiologists guidelines and hospital policy [11, 15]. 

Sedation delivery is divided between nursing administered and anesthesiologist delivered. Nursing-administered sedation is limited to the oral route with midazolam or chloral hydrate only. Only ASA 1 and 2 patients over the age of one month are allowed to be sedated by nurses who must be able to visualize the patient throughout. Patients who do not meet the above criteria are referred to an anesthesiologist for direct management. A review of outcome supports the screening process in the majority of cases: of all procedures which are under the direct care of a nurse, anesthesiologist assistance is required in 6.5% of the cases. Eighty percent of all procedures are triaged to anesthesiologist management with propofol, and the remaining 20% are sedations that are delivered by nursing. 

The most frequent adverse event recorded was a decrease in oxygen saturation, which occurred in 132 cases (1.5% of all cases), all under the care of an anesthesiologist. All these children were sedated either in the oncology clinic (35 patients) (where some refused to accept an oxygen mask before sedation) or for flexible bronchoscopy (97 children), where decreases in oxygen saturation are frequent. All these children had received propofol as the sole sedative agent. The oxygen saturation recovered spontaneously in 74 children and after an increase in oxygen flow in the remaining 58 children. Postsedation vomiting was noted in 6 children (0.07% of all cases) on arousal and resolved spontaneously with no respiratory or other complications and without the need for hospital admission. Finally, cardiac arrhythmia that did not require specific treatment was recorded in 12 children undergoing cardiac angiography.

### 2.2. Gastroenterologist Directed Sedation Models: From the United States to South America

Gastroenterologists in the United States and Europe have lead the way in establishing guidelines and presenting outcomes for gastroenterologist-administered and/or supervised sedation of adults [6, 16, 17]. The literature on pediatric sedation performed by gastroenterologists for upper and lower endoscopy is limited. In the United States, fentanyl and midazolam remain common agents administered via the intravenous route [18]. The addition of capnography, although not required by the American Society of Gastroenterologists, is recognized as a useful means of identifying and managing alveolar hypoventilation prior to the occurrence of oxygen desaturation [18]. 

 Pediatric gastroenterologists in the United States have described the administration of ketamine as efficacious for gastrointestinal sedation, with an accompanying 9.5% incidence of transient laryngospasm [19]. In Brazil, 78.6% of all pediatric endoscopies at a large hospital described the use of midazolam and meperedine sedation administered under the auspices of pediatricians or gastroenterologists. The remainder of the procedures, approximately 20%, was performed by anesthesiologists under general anesthesia [20]. 

 Nursing-Administered Propofol Sedation (NAPS) or Nonanesthesia-Administered Propofol Sedation (NAAPS) are mnemonics which refer to the administration of propofol by qualified nurse(s) who operate under the direction of a nonanesthesiologist physician. To date, this technique has only been applied for adult sedation. Although NAPS/NAAPS is an accepted method of propofol administration by the American Society of Gastroenterologists, its administration by nurses is prohibited or restricted by many State Registries of Nursing within the United States. For example, on October 13, 2005 the Minnesota Board of Nursing issued a statement which supported the administration of propofol by registered nurses but specified that the nurse also has the prerogative to decline delivery should it be perceived as unsafe in the particular circumstance [21]. 

NAPS is administered via algorithms, all of which were intended for patients over 12 years of age [22, 23]. It is important to recognize that NAPS was not designed with the intent for pediatric application, because most adult sedations are moderate, while most pediatric sedations are deep. Obviously, this difference significantly changes the risk of adverse events. A prospective cohort study of 27,061 adults evaluated the need for airway rescue with NAPS in two ambulatory GI settings which administered propofol consistent with NAAPS guidelines. Propofol was administered by the endoscopy nurse and supervised by the endoscopist. Monitoring consisted of pulse oximetry and clinical assessment. A mean propofol dose of 161 mg (range 50–650 mg) was used for endoscopic gastroduodenoscopy and 116 mg (range 30–500 mg) with 25 mg of meperidine administered for colonoscopy. The target was moderate-to-deep sedation. It is interesting to notice that less propofol was used for “lower” endoscopies, because meperidine was added as an adjuvant. Oxygen saturation fell below 90% in 2.3% of the adults and 6 patients required brief positive pressure ventilation [12, 17]. Only 23% of all the patients had oxygen before the procedure. 

A recent study of 498 nurse administered propofol sedations for bronchoscopy (18–86 years of age) reported similar results. 1-2 mg IV midazolam and 25–50 mcg IV fentanyl is administered prior to a 20–40 mg IV propofol bolus. 10–20 mg IV propofol is administered every minute to maintain adequate sedation. The propofol is titrated to the sedation requirements of the procedure. The average propofol dose was 3.13 mg/kg (range 0.12–20 mg/kg). Every patient received supplemental oxygen during the procedure. Overall, there was a 6.6% incidence of sedation related adverse events. 2.8% were reported as major adverse events, which included pulmonary hemorrhage (1.2%), hypoxia/respiratory failure (0.8%), bronchospasm (0.2%), airway obstruction by tumor (0.2%), stridor (0.2%), and pneumothorax (0.2%) [24]. 1.2% of these major events were classified as likely to be sedation-related. There was no sedation-related death. This study was not randomized. The safety of NAPS may have been confounded by the supplemental fentanyl and midazolam. 

The worldwide safety experience of endoscopist-administered propofol sedation now exceeds 460,000 patients [25–27]. Additional studies are warranted in order to validate the safety of NAPS in varied clinical settings, for patients of varied ages and medical conditions. To the best of our knowledge, the application of NAPS for pediatric sedation is not being practiced at this time nor is it supported by any specialty society worldwide, for many reasons (deeper sedation is usually required in children, their airways are narrower, and their time to reaction to an adverse event is shorter).

Although the adult literature cites propofol administration by nurses and gastroenterologists, the pediatric literature describes only anesthesiologist-delivered propofol for pediatric gastrointestinal procedures. The risk of respiratory depression, apnea, and cardiovascular instability in addition to the narrow therapeutic window between spontaneous ventilation and apnea has deterred pediatric gastroenterologists and other nonanesthesia care providers from using it for pediatric endoscopy [28–30].

### 2.3. Hospitalist-Delivered or Supervised Sedation in the United States

Hospital medicine is an evolving specialty which for pediatrics, is represented by pediatricians, emergency medicine or intensive care medicine physicians. The majority of pediatric hospitalists are pediatricians who are committed to a hospital-based practice. Pediatric hospitalists have developed sedation programs in collaboration with their hospital's Department of Anesthesia. 

At St. Louis, a pediatrician-delivered propofol sedation program sets the standard for organization, safety, and comprehensive services. Recent oral presentations at the Pediatric Academic Society meeting at Vancouver, May 1–4, 2010 presented the outcomes of their nonanesthesiologist-delivered sedation program (written communication). Under the direction of Dr Doug Carlson, the Chief of Pediatric Hospital Medicine at the Children's Hospital, St. Louis of Washington University, pediatricians undergo rigorous didactic and practical training in sedative administration and airway management. The hospitalists deliver over 2,000 sedation per year, mostly ketamine based. 

At this program, there is a three-tiered system of pediatrician delivered sedation, each tier of which requires specified training. The first tier provides sedation services in the emergency department, primarily utilizing ketamine or nitrous oxide. Training for this tier consists of a two-hour didactic orientation with continuing hands-on experience. The second tier provides sedation throughout the hospital and includes the emergency department, ambulatory areas, and inpatient areas both during the day and as needed overnight. Pediatricians who provide second-tier service may use the agents of the first-tier in addition to pentobarbital and dexmedetomidine. Training for this tier requires a provision of first-tier services for a minimum of a year in addition to five days of operating room (OR) training in sedation administration and airway management with an anesthesiologist. The third-tier sedation service builds upon the 1st and 2nd tier with the additional credentialing to provide deep sedation with propofol. Propofol credentialing requires a three-hour didactic session followed by ten days of OR training under the auspices of an anesthesiologist and the completion of 25 supervised propofol sedations. In order to maintain certification to deliver propofol, the pediatricians must administer a minimum of 50 propofol sedations per year, always with the immediate availability of an anesthesiologist if requested.

### 2.4. Emergency Medicine-Delivered Sedation Programs

In the United States, pediatric emergency medicine is a specialty of its own. Although not yet a recognized specialty in other countries, the emergency medicine physicians have lead the way in providing pediatric sedation. Historically, as early as the 1980s, the delivery of sedation by emergency medicine physicians was limited to the emergency department (ED) site only [31, 32]. 

Over the past decade, some of these emergency medicine physicians have established sedation services throughout the hospital, primarily in the Department of Radiology for imaging studies [33–35]. The delivery or supervision of moderate-to-deep sedation by emergency medicine physicians is a growing practice for many reasons: the foremost reason is that these physicians already have sedation skills and are proficient in airway management and cardiovascular resuscitation. Many children's hospitals have established formal sedation training processes for credentialing emergency medicine physicians in pediatric sedation. This training has included an educational program which involves didactics, reading material, and successful completion of a multiple choice test for all emergency medicine physicians and nurses involved in sedation [36–40]. 

The emergency medicine specialty has made valuable contributions to the sedation literature, particularly with respect to ketamine delivery, the introduction of new sedative agents and sedation outcomes. A meta-analysis of pooled individual-patient data from 32 ED studies examined the clinical variables which predict airway and respiratory adverse events with ketamine administration by emergency medicine physicians. In 8,282 pediatric ketamine sedations, the overall incidence of airway and respiratory adverse events was 3.9%, with the following significant independent predictors: younger than 2 years (odds ratio [OR] 2.00), aged 13 years or older (OR 2.72), high intravenous dosing (initial dose of 2.5 mg/kg or total dose of 5.0 mg/kg; OR 2.18), coadministration of anticholinergic (OR 1.82), and coadministration of a benzodiazepine (OR 1.39). Oropharyngeal procedures, underlying physical illness (American Society of Anesthesiologists class 3), and route of administration (intravenous versus intramuscular) did not predict adverse outcome [41]. In another consecutive case series of 1,022 children, Green et al. report that ketamine at doses of 4 to 5 mg/kg intramuscularly produced adequate sedation in 98% of children. They reported airway complications in 1.4% of patients that included laryngospasm, apnea, and respiratory depression, all of which were quickly identified and treated without intubation or sequela. Emesis occurred in 6.7% without evidence of aspiration [42]. 

Historically, ketamine, narcotics, nitrous oxide, and benzodiazepines were the agents of choice in the ED. Ketamine has been administered alone or in combination with other sedatives. The published outcomes have been important in establishing the safety of emergency medicine sedation practice. In a randomized controlled trial in 260 children aged 5 to 15 years, Kennedy et al. found that a ketamine and midazolam combination was safer and more efficacious than a fentanyl and midazolam combination for sedation in orthopedic procedures. Hypoxia, while children breathed room air, occurred in 6% of patients receiving ketamine and midazolam versus 20% of patients in the fentanyl and midazolam group [43]. 

Over the past decade, propofol has been gaining widespread interest. Although propofol is considered by the FDA to be an anesthetic agent, the American College of Emergency Physicians has included it in their sedation guidelines [7]. There is a growing body of evidence supporting the safe use of propofol for procedural sedation by emergency physicians. A review presented adverse events following propofol sedation of children following an opioid premedication, prior to undergoing orthopedic reduction in the emergency department. All children received supplemental oxygen (1 L/minute by nasal cannula) and continuous capnography and had depth of sedation assessed every 2 minutes. Adverse airway or respiratory events with intervention occurred in 14 of the 125 enrolled children (11%): jaw thrust in 4/125, the need for increased supplemental oxygen in 6/125, and bag-valve-mask ventilation in 4/125. All interventions required were brief (<30 seconds). Capnography was successful in detecting apnea before clinical examination or pulse oximetry in all 5 occurrences and similarly first detected airway obstruction in 6 of the 10 occurrences. The median maximal modified Ramsay score was 6 (range 3 to 8), that is, deep sedation [44]. 

 In another prospective observational study performed in the ED, propofol-induced procedural sedation was reported to have the lowest rate of respiratory depression when compared with methohexital, fentanyl/midazolam, and etomidate [45]. There were no significant complications.

 Regardless of which agents and which route of delivery are chosen for the delivery of sedation by emergency medicine physicians, the outcomes parallel those of other specialties. A review of a pediatric emergency medicine-staffed sedation service for radiological imaging studies showed that of 923 sedations, overall there was a 10% incidence of adverse events. The majority of the sedations included pentobarbital, fentanyl, midazolam, and/or chloral hydrate. 55 patients received propofol alone. There was a small 0.76% incidence of major adverse events (significant hypoxemia, apnea, laryngospasm, and stridor) which required intervention that may have included repositioning, brief positive pressure ventilation, oral or nasal airway, supplemental oxygen, or vigorous stimulation. Sedation failed to achieve adequate conditions in 17 (1.8%). There was no incidence of endotracheal intubation or cardiopulmonary resuscitation with pharmacologic intervention [33]. 

In Memphis, Tennessee, a university-affiliated group of pediatric emergency physicians provide sedation services to a radiology department during weekdays at a freestanding urban children's hospital. Of 1285 patient encounters, deep sedation was provided to 1027 children with pentobarbital (midazolam, fentanyl, or both added to pentobarbital if needed) in 65% of cases, propofol in 31%, and ketamine (with or without midazolam) in 4%. 258 children received moderate sedation with chloral hydrate (86%) and 14% received oral diazepam. Procedural sedation times for the most frequently performed imaging studies ranged from 5 to 183 minutes, with a 99.1% incidence of successful imaging studies. The incidence of adverse events was extremely low: 3 children (0,2%) had adverse events which included oxygen desaturation <90% which required in one child brief positive pressure ventilation and hypotension requiring intravenous crystalloids [35]. Other studies support these outcomes and demonstrate that both moderate and deep sedation can be safe and effective when properly administered by experienced emergency physicians [46, 47]. 

As the emergency department continues to provide sedation services in other areas of the hospital, there may arise a difference of opinion between the emergency medicine physicians and their anesthesia colleagues over a variety of issues. The first issue is that of NPO (nil per os) standards. The emergency medicine physician is frequently accustomed to deliberating the risks versus benefits of providing sedation to children who present in an emergent situation. These situations require balancing the emergent/urgent need to deliver sedation for a procedure against the failure to adhere to ASA and AAP guidelines and the possible aspiration risk associated with a curtailed NPO time [11, 15]. 

The emergency medicine literature has provided large studies which review the outcome of sedating children outside of the NPO recommendations. The largest study to date reviewed 1014 patients for whom fasting status was available for 905 (89%) patients. Of these 905 patients, 509 (56%) did not meet fasting guidelines as suggested by the American Academy of Pediatrics and the American Society of Anesthesiologists. In this group, there were no episodes of aspiration. Seventy-seven adverse events occurred in 68 (6.7%) of the 1,014 patients. All adverse events were minor and successfully treated. These adverse events occurred in 32 (8.1%) of 396 patients who met and 35 (6.9%) of 509 patients who did not meet fasting guideline Emesis occurred in 15 (1.5%) patients. There were no episodes of aspiration [48]. But much more larger studies are required to accurately validate the incidence of these rare adverse events [42, 49]. Using careful triage and evaluation, including assessment of the urgency of the required procedure, emergency medicine physicians have supported their practice of delivering sedation outside of NPO recommendations when appropriate: they use mostly ketamine which relatively preserves the protective reflexes. Also, there is a lack of airway manipulation with an endotracheal tube. All together, that may reduce the risk of aspiration, compared to general anesthesia. Another factor that can influence their decision, is the administration of opioids, which can delay gastric emptying. 

Another area of controversy is the utility of supplemental oxygen delivery during sedation. In a 2007 review of emergency medicine-delivered sedation, the role of supplemental oxygen as a standard was reviewed. Supplemental oxygen did not reduce (or trend toward reducing) the incidence of hypoxia in patients moderately sedated with midazolam and fentanyl. With deep sedation, supplemental oxygen was determined to mask transient desaturation which can occur after a sedative drug bolus [50, 51]. 

### 2.5. Critical Care Specialists and Sedation Models

Some sedation models utilize intensive care medicine physicians to administer and provide pediatric procedural sedation out of the intensive care unit. One such model is at the Children's Hospitals and Clinics of Minnesota, Minneapolis. Over a 3-year period, they described the outcome of 7304 propofol elective sedations which were administered by critical care physicians and advanced practice nurses, under the auspices of an anesthesiologist. The most common procedures were diagnostic radiological imaging studies (MRI, CT, and nuclear medicine), short oncologic procedures (lumbar punctures, bone marrow biopsies, and intrathecal chemotherapy) and neurological testing which includes electroencephalograms, evoked potentials and hearing tests. All patients received supplemental oxygen. They report a 2.9% incidence of oxygen desaturation <85%, hypotension in 31.4% (drop of systolic BP of ≥25 mm Hg from baseline), intubation in 0.03%, and the need for brief positive pressure ventilation in 0.37%. There were no failed sedations and no cardiopulmonary resuscitation [52]. The outcomes rivaled those published by Cravero et al. of 49, 836 propofol sedations provided by physician and nurse providers of different specialties. Almost half of the sedation care providers were identified as intensive care physicians. This consortium of sedation care providers from multi-institutions reported brief desaturation <90% in 7.16%, cardiac arrest in  .02%, intubation in  .53% and positive pressure ventilation in 5.13% [1]. Further studies are needed to determine whether there is a difference in outcome between the different specialists administering propofol, and between fasted and nonfasted patients. Both of these studies are confounded by different definitions of adverse events, a varied patient population and lack of uniform propofol protocols which would have standardized delivery regimens and provided a more accurate means of comparison.

### 2.6. Nursing Delivered

A large model of nursing delivered pediatric sedation is at Boston Children's Hospital, within which there are approximately 7,000 nursing-administered sedations performed annually. Half of these sedations are delivered in the Department of Radiology, 25% in the Emergency Department and the remaining 25% are scattered throughout the hospital (oncology, dental, gastroenterology, and cardiology). Within the institution, the Department of Radiology sets the standard for a protocol-driven sedation program, administered by specialized nurses under the direct supervision of sedation-designated anesthesiologists. These anesthesiologists represent a small, core group of physicians who are committed to safe, efficacious sedation delivery as well as to the collection of reliable Quality Assurance (QA) data. The QA data sheets are designed and tailored to each sedation area as well as to the sedation agent. This QA data is reviewed and analyzed monthly and is the essence of the sedation program, guiding the evolution of sedation practice. As the sedation program has evolved, the older sedatives such as pentobarbital and chloral hydrate have been largely replaced with dexmedetomidine and ketamine.

A review of 16,467 elective sedations delivered by radiology nurses at Boston Children's Hospital reported a total of 70 (0.4%) pulmonary adverse events: 58 oxygen desaturations (<5% of baseline for over 60 seconds), 2 pulmonary aspirations (no clinical sequelae), 10 airway resuscitations (brief positive pressure mask ventilation), and 0 (0.0%) cardiovascular events. There was no cardiac arrest and no need for intubation. Single sedation agents were associated with a lower risk than the administration of multiple agents (*P* < .001) [53]. 

## 3. Sedation Guidelines and Recommendations: A Global Overview

 The challenge facing sedation care providers is the need to balance the delivery of safe and effective sedation while adhering to the sedation guidelines of one's specialty's society. The sedation guidelines are not all consistent between specialty societies. This paper will compare the sedation guidelines of existing specialty organizations as well as of some institutions, highlighting the similarities, differences and opposing views on areas of particular interest. 

A global look at sedation guidelines reveals that there is lack of consistency not only between the specialties within a single continent, but also between the continents. These guidelines differ not only with respect to appropriate medications, routes of delivery, NPO status, and physiological monitoring requirements, but also with respect to the appropriate skill sets of the sedation care provider who delivers different levels of sedation. We will review the notable sedation guidelines of notable adult and pediatric specialty societies (anesthesia, dental medicine, emergency medicine, and gastroenterology) both within the United States and abroad. We will identify the important and controversial differences between the guidelines.

### 3.1. The American Academy of Pediatrics (AAP) [54, 55] 

#### 3.1.1. Overview

In 1983, after three children died in a single dental office, the AAP charged the Section on Anesthesiology with the responsibility of developing guidelines for the sedation practice of children by nonanesthesiologists. In 2002, a clarifying addendum to the AAP guideline was published [55]. Subsequently, the ASA revised the document which defined the sedation levels within the sedation continuum, descriptors which were adopted by the Joint Commission of Accreditation of Healthcare Organizations (Joint Commission) [56]. These guidelines were designated for children who received sedation in all in and out of the hospital-venues, including private offices. This addendum retired the phrase “conscious sedation” in preference for depths of “sedation/analgesia” that included minimal, moderate, and deep sedation. They emphasized that sedatives were only to be administered under medical supervision (no home prescriptions) and only by “individuals skilled in airway management and cardiopulmonary resuscitation”. These guidelines introduced the important concept of ensuring that sedation care providers were skilled and trained in “patient rescue” [56]. 

In 2006, the guidelines were again updated to specify that sedation must be administered under appropriate medical supervision throughout all aspects of the sedation and recovery period; after careful presedation evaluation for underlying medical or surgical conditions and after appropriate fasting (NPO) for elective procedures. The NPO status must be considered in context of the need to perform the procedure when sedation is required urgently. Those who require sedation urgently may have NPO status waived after a careful assessment of the risk and benefits associated with delaying the procedure. The importance of a focused airway examination for large tonsils or anatomic airway abnormalities was identified along with the need for providers to have a clear understanding of the pharmacology of the sedatives and appropriate emergency skills, pharamacologic agents, and equipment needed for rescue. An emergency cart must be immediately accessible and stocked with age- and size-appropriate drugs and equipment to resuscitate a child of any size. Monitoring devices should include electrocardiography (ECG) machines, pulse oximeters (appropriate selection of sizes), and defibrillators [55]. End-tidal carbon dioxide monitors are very useful in situations where the child is not directly observed like in the MRI.

#### 3.1.2. Summary of Important Recommendations

**NPO Guidelines**

 Clear liquids: 2 hours: include water, fruit juices without pulp, carbonated beverages, clear tea, black coffee
 Breast milk: 4 hours
 Infant formula, Nonhuman milk
 Light meal and solid food: 6 hours

**Credentials Required to Administer Deep Sedation**

- There must be 1 person available whose sole responsibility is to constantly observe the patient's vital signs, airway patency, and adequacy of ventilation and to either administer drugs or direct their administration.
- At least 1 individual, trained and competent to provide advanced pediatric life support, airway management, and cardiopulmonary resuscitation, must be present.

**Guidelines for Propofol Administration**

There is no statement or recommendations.

**Recommendations for Capnography**

Not required but encouraged, particularly in situations where other means of assessing the adequacy of ventilation are limited [57–59].

The ASA House of Delegates on October 21, 2009, issued a statement on respiratory monitoring during endoscopic Procedures. The statement advised that capnography be considered when propofol alone or in combination with opioids and/or benzodiazepines be used for sedation [60].

### 3.2. American Society of Anesthesiologists (ASA) [61] 

#### 3.2.1. Overview

The American Society of Anesthesiologists (ASA) has developed “Guidelines for Sedation and Analgesia by Nonanesthesiologists which emphasize the importance of the sedation continuum in following the depths of sedation from minimal sedation to general anesthesia [56]. 

#### 3.2.2. Summary of Important Recommendations

**NPO Guidelines**

In emergency situations, when preprocedure fasting is not practical, the target level of sedation should be modified (i.e., less sedation should be administered) for moderate sedation as well as deep.

 Clear liquids: 2 h
 Breast milk: 4 h
 Infant formula: 6 h
 Nonhuman milk: 6 h
 Light or solid meal: 6 h

**Credentials Recommended to Administer Deep Sedation**

Privileges to administer deep sedation should be granted only to practitioners who are qualified to administer general anesthesia or to appropriately supervise anesthesia professionals [62]. This individual should have no other responsibilities except to deliver sedation and monitor the patient throughout.

**Guidelines for Propofol Administration [63]**

All patients who receive propofol (or methohexital) should receive care consistent with deep sedation. Accordingly, practitioners administering these drugs should be qualified to rescue patients from any level of sedation, including general anesthesia.

**Recommendations for Capnography**

Capnography should be considered, but is not required, for all patients receiving deep sedation and for patients whose ventilation cannot be directly observed during moderate sedation.

**Recommendations for Physiologic Monitoring**

- Pulse oximetry with appropriate alarms is required.
- Ventilatory function should be continually monitored by observation or auscultation.
- Blood pressure should be determined before sedation/analgesia is initiated and measured at 5-min intervals during the sedation, unless such monitoring interferes with the procedure.
- Electrocardiographic monitoring required with all deep sedation and with those who have cardiovascular disease or are at risk of dysrhythmias.

**Recommendations for Oxygen Delivery**

Supplemental oxygen should be used during deep sedation to reduce the frequency of hypoxemia.

### 3.3. Joint Commission of Hospital Accreditation in United States [64, 65] 

#### 3.3.1. Overview

The JCAHO 2004 Comprehensive Accreditation Manual for Hospitals was intended to set the standards for sedation and anesthesia care for patients in any setting. Standard PC  .03.01.01 requires that a sufficient number of staff, in addition to the person performing the procedure, be present to perform the procedure, monitor, and recover the patient. The person administering the medication must be qualified to monitor the patient as well as manage whatever level of sedation or anesthesia is achieved, either intentionally or unintentionally [64]. 

These guidelines were meant to be inclusive of all levels of sedation as well as general, spinal, or regional anesthesia. They specified that in order to minimize complications, the appropriate drug(s) and dosages must be chosen, monitored, and administered in the proper setting, and a patient evaluation should be performed before, during, and after their use.

**Credentials Recommended to Administer Deep Sedation**

The anesthesia care standards require that the individuals who are “permitted” to administer sedation are able to rescue patients, independent of a code team, from whatever level of sedation or anesthesia is achieved either intentionally or unintentionally, for example, when the patient slips from moderate into deep sedation or from deep sedation into full anesthesia. Each organization is free to define how it will determine that the individuals are able to perform the required types of rescue. The Joint Commission does not specify the training or equipment for proper rescue.

**Guidelines for Propofol Administration**

The Joint Commission standards do not identify specific medication. Rather, they expect that the appropriate medication be chosen for the intended level of sedation desired.

**Expectations for Patient Assessment**

Joint Commission standards require that the patient is reevaluated immediately (either on the procedure table or in the moments prior to administering sedation) before administering moderate or deep sedation or before the induction of anesthesia. Typically, the assessment includes vital signs, status of the airway, and response to any preprocedure medications [66].

### 3.4. American Association of Pediatric Dentistry/American Dental Association [4] 

#### 3.4.1. Overview

In 2006, the American Dental Association (ADA) published guidelines for the safe and effective sedation by appropriately educated and trained dentists. For children 12 years of age and under, the ADA supports the use of the American Academy of Pediatrics/American Academy of Pediatric Dentists Guidelines for Monitoring and Management of Pediatric Patients During and After Sedation for Diagnostic and Therapeutic Procedures [55]. 

These guidelines apply to pediatric dental patients and include two paragraphs which identify areas which are especially challenging: the sedation of the special needs patients and management of emergency situations. These guidelines recognized that if the dental patient undergoing deep sedation or general anesthesia is mentally and/or physically challenged, it may not be possible to have a comprehensive physical examination or appropriate laboratory tests prior to administering care. In these situations, the dentist responsible for administering the deep sedation or general anesthesia should document the reasons preventing the recommended preoperative assessment prior to administering sedation. 

These guidelines did not require intravenous access for all patients. Rather, they condoned that in selected circumstances, deep sedation or general anesthesia may be utilized without establishing an indwelling intravenous line. These selected circumstances may include brief procedures or situations in which intravenous access is not possible.

The guidelines also reiterated those of the Joint Commission and AAPD with respect to emergency situations. The dentist responsible for the sedation accepts responsibility for the management of the sedation/anesthetic as well as for the identification and treatment of sedation/anesthesia related emergencies. Most important, this dentist assumes responsibility for ensuring the adequacy of the facility and staff and for providing the equipment, drugs, and protocols for patient rescue.

These guidelines differed from other guidelines in that they specifically identified nitrous oxide as an agent which could be used alone or in combination with other sedatives in order to achieve sedation or anesthesia.

**NPO Recommendations**

There are no specific recommendations. They advise that preoperative dietary restrictions must be considered based on the intended depth of sedation or anesthesia.

**Credentials Recommended to Administer Deep Sedation**

A minimum of three individuals must be present: one dentist who is credentialed to administer deep sedation or anesthesia and 2 additional personnel who have current certification of successfully completing a Basic Life Support (BLS) Course for the Healthcare Provider.

The dentist must be qualified to administer the deep sedation or general anesthesia. There are 2 requirements to qualify. The first qualification requires successful completion of an advanced education program on the administration and management of deep sedation or anesthesia, which must be accredited by the ADA Commission on Dental Accreditation. The second requirement is a current certification in both Basic Life Support for Healthcare Providers and Advanced Cardiac Life Support (ACLS) or an appropriate dental sedation/anesthesia emergency management course.

The dentist administering deep sedation or general anesthesia must remain within the facility until the patient meets discharge criteria (or is discharged) and must monitor the patient continuously until the patient meets the criteria for recovery.

These guidelines are unique to the others, in that they allow the dentist to provide the deep sedation/anesthesia to also perform the procedure. In these circumstances, one of the additional appropriately trained team members must be designated for patient monitoring.

**Guidelines for Propofol Administration**

There is no discussion of propofol in these guidelines.

**Recommendations for Capnography**

 Intubated patients: Capnography required.
 Nonintubated patients: breath sounds must be assessed via auscultation or capnography must be continually monitored.

### 3.5. American College of Emergency Physicians (ACEP) [7] 

#### 3.5.1. Overview

Similar to the ASA guidelines, the ACEP guidelines apply to all patients, adults and children who receive sedation. They recognize that sedation is a continuum and maintains that practitioners should possess the skills required to rescue a patient from one level beyond the intended level of sedation. These skills are expected to include a competence in cardiovascular resuscitation and airway management which should include a patient who has achieved general anesthesia. The ACEP guidelines consider these skills to be a fundamental part of the emergency medicine training curriculum and inclusive of the training required of all board-certified emergency physicians. These guidelines are comprehensive and include and update some previously unaddressed issues and recommendations [67].

**Credentials Recommended to Administer Deep Sedation**

The ACEP guidelines consider that a board-certified emergency physician is qualified to administer deep sedation. Should this physician also be performing the procedure, the guidelines specify that a qualified support person be present for continuous monitoring of the patient.

**NPO Recommendations**

The guidelines state that although “recent food intake is not a contraindication for administering procedural sedation and analgesia, the emergency physician must weigh the risk of pulmonary aspiration and the benefits of providing procedural sedation and analgesia in accordance with the needs of each individual patient [7].” The NPO recommendations are based upon preliminary, inconclusive or conflicting evidence and state that “recent food intake is not a contraindication for administering procedural sedation and analgesia, but should be considered in choosing the timing and target level of sedation [7].”

**Capnography Recommendation**

ETCO2 monitoring is not required but may allow more rapid identification of hypoventilation than pulse oximetry alone [58].

**Pulse Oximetry Recommendations**

The ACEP guidelines are unique in that unlike the ASA or AAP guidelines, pulse oximetry is not mandatory. The guidelines advise that pulse oximetry may not be necessary when the patient's level of consciousness is minimally depressed and verbal communication can be continually monitored. Pulse oximetry is recommended, however, when there is an increased risk of developing hypoxemia, such as when high doses of drugs or multiple drugs are used, or when treating patients with significant comorbidity.

**Guidelines for Propofol Administration**

The ACEP guidelines specify that propofol can be safely administered for procedural sedation and analgesia in the emergency department.

### 3.6. American Society of Gastroenterologists [5, 12] 

#### 3.6.1. Overview

The Standards of Practice Committee of the American Society for Gastrointestinal Endoscopy (ASGE) prepared these guidelines in conjunction with a search of the medical literature using MEDLINE and PubMed databases. These guidelines apply to all patients, both adults and children, who receive sedation. The ASGE has approved the ASA guidelines for sedation by nonanesthesiologists and assert that an anesthesia specialist is not cost effective for average-risk patients undergoing routine upper and lower endoscopic procedures. 

The guidelines recommend that with an intravenous benzodiazepine and opioid combination, adequate and safe sedation can be achieved in most patients undergoing routine esophagogastroduodenoscopy and colonoscopy. Others drugs such as droperidol can be used.

**Credentials Recommended to Administer Deep Sedation**

Deeper levels of sedation may be considered for longer and more complex procedures or for those who have been difficult to manage with moderate sedation and are anticipated to be poorly responsive to sedatives. Indications may include those patients who have had long-term use of narcotics, benzodiazepines, and alcohol. Deep sedation requires at least 1 person who is dedicated to the uninterrupted monitoring of the patient and is qualified in advanced life support skills needed to rescue a patient who becomes unresponsive, unable to protect the airway, or who loses spontaneous respiratory or cardiovascular function.

**Recommendations for Pulse Oximetry**

The ASGE follows the recommendations of the ASA and recommends that pulse oximetry be used during all endoscopic procedures [61, 68].

**Recommendations for Propofol Administration**

Propofol can be safely and effectively given by nonanesthesiologist physicians and nurses provided they have undergone appropriate training and credentialing in administration and rescue from potential pulmonary and cardiovascular complications. The guidelines state that clinically important benefits of propofol in average-risk patients undergoing upper endoscopy and colonoscopy have not been consistently demonstrated with regard to patient satisfaction and safety.

**NPO Guidelines**

The ASGE follows the ASA guidelines:

- NPO 2 hours clear liquids.
- NPO 6 hours after light meals.

**Recommendations for Capnography**

Capnography is not required, although the ASGE indicates that integrating it into patient monitoring protocols may improve safety, acknowledging that there is insufficient evidence to support its use during routine upper and lower endoscopic sedation [69–72].

The ASGE guidelines cite the ASA guidelines in stating that capnography ‘‘should be considered for all patients receiving deep sedation and for patients whose ventilation cannot be observed directly during moderate sedation [61].”

### 3.7. The Scottish National Guidelines [9] 

#### 3.7.1. Overview

In Scotland, the sedation guidelines are meant to encompass minimal and moderate sedation only. Nonanesthesiologist delivered sedation is restricted and nurse administered sedation is only condoned with strict protocols, comprehensive backup and a comprehensive clinical governance and risk management framework. Deep sedation is given the same considerations as a general anesthetic and requires an identical standard of care.

These guidelines specify that children be sedated as proximal as possible to the procedure location and never at home. Patient assessment is important for these Guidelines in that they guide the choice of sedation care provider. Specifically, abnormal airway, sleep apnea, or respiratory tract infection are contraindications for sedation by nonanesthesiologist personnel, and require an anesthesiologist. Precautions are recommended with neonates, premature babies, emergency cases, or for children who are receiving narcotics.

Sedation practice in Scotland offers a unique viewpoint on the role of the child and parent in the sedation process. In 1995, the Child Scotland Act specified that an informed consent be obtained from the child when appropriate. The presence of the parents is recommended during the sedation, in hopes of providing emotional support.

**NPO Guidelines**

 Clear fluids: 2 hours
 Breast milk: 4 hours
 Formula or bottle milk: 6 hours

Nitrous Oxide, if used alone, does not require any NPO status

Emergency Procedures: if NPO status is unable to be met, general anesthesia recommended.

**Guidelines for Administration of Deep Sedation**

In the United Kingdom, deep sedation is considered to be a part of the spectrum of general anesthesia and administration should be limited to anesthesiologists. Those who administer deep sedation should not be performing the procedure.

**Recommendations for Propofol Administration**

Propofol is considered to be a general anesthetic and administration should be restricted to anesthesiologists.

**Recommendations for Capnography**

Capnography is recommended but not compulsory.

### 3.8. South African Society of Anaesthesiologists [10]

In South Africa, separate adult and pediatric sedation guidelines exist for the South African Society of Anaesthesiologists. The pediatric guidelines were written by Dr A. Reed, Dr R. Gray, Dr M. de Kock, Prof J. Thomas, Dr J. Piercy, and Prof J. Roelofse and shared with the authors (written correspondence).

#### 3.8.1. Overview

The South African Society of Anaesthesiologists will publish in 2010 the Paediatric Procedural Sedation and Analgesia (PSA) Guidelines. These guidelines are intended for painful and nonpainful procedures but are not meant for sedation of children in the intensive care unit, under conditions of palliative care, for sedation at home, for “night sedation” or for preoperative sedation. These guidelines distinguish sedation in the hospital setting from sedation outside the hospital setting. The airway exam is identified as an essential requirement of the presedation evaluation and is used to differentiate those children who are appropriate for sedation in settings outside of the hospital from those who require sedation in a hospital. Specific airway factors which include but are not limited to retropharyngeal masses, Mallampati>2, stridor, large tonsils, obstructive sleep apnea, syndromic features (large tongue, micrognathia and abnormal ears) and limited neck mobility should exclude a patient from receiving sedation outside of the hospital setting. 

These guidelines identify two different sedation techniques—“simple” and “advanced”. Simple/basic sedation uses a single agent (not a combination of single agents), typically an oral/transmucosal/rectal drug (e.g. small dose oral benzodiazepine) or inhalation of nitrous oxide (N_2_O) in at least 50% oxygen. It requires appropriate NPO status and cannot progress beyond the administration of one sedative agent. Advanced sedation encompasses a technique which administers multiple sedatives, uses the intravenous route or an inhalation anesthetic or nitrous oxide in a concentration of greater than 50%.

**NPO Guidelines**

 Clear fluids: 2 hours
 Breast milk: 4 hours
 Formula and solid food: 6 hours

When N_2_O is used alone (50%), no fasting is necessary.

In urgent cases, when NPO guidelines are not met, a general anesthetic with rapid sequence induction is encouraged.

**Recommendations for Deep Sedation**

Considered to be part of the spectrum of general anesthesia and should be administered only by trained anesthesiologist.

**Recommendations for Propofol Administration**

Propofol should only be administered by experienced seditionist skilled in airway management of children. Capnography is highly recommended with propofol. Targeted controlled infusions are highly recommended with propofol in order to avoid the risk of respiratory depression with repeat bolus injections and infusions.

**Recommendations for Capnography**

Capnography is recommended for advanced sedation. If capnography is not available, a precordial stethoscope is recommended.

### 3.9. Saudi Arabia (National Guards Health Affairs) [8] 

#### 3.9.1. Overview

The pediatric sedation guidelines In Saudi Arabia are based upon the American Society of Anesthesiologists guidelines. These guidelines apply to sedation by non anesthesiologists in areas of dental medicine, pediatrics, cardiology, obstetrics and gynecology, oncology, and gastrointestinal medicine. These guidelines indicate that future consideration will be given to permit non-anesthesiologsts to deliver fos-propofol.

**NPO Guidelines**

All patients

 Clear fluids: 2 hours
 Breast milk: 4 hours
 Formula and bovine milk: 6 hours
 Meal: 8 hours

**Recommendations for Deep Sedation**

The sedation provider will be solely responsible for the monitoring and care of the patient, and not for performing the procedure.

The process of credentialing requires:

- documented attendance at an approved sedation by nonanesthesiologist course and
- a minimum current certification in BLS, or preferably ACLS, issued by National Guard Health Affairs, the Saudi Heart Association or the American Heart Association. For pediatric sedation, a current PALS certification is required.

**Recommendations for Propofol**

Propofol administration is restricted to anesthesiologists when used for procedural sedation in nonintubated children.

**Recommendations for Capnography**

No specific recommendations.

Oxygen saturation, heart rate, blood pressure, respiratory rate, and level of consciousness are required data elements.

## 4. Discussion

The need for pediatric sedation has increased over the past decade, likely paralleling the increasing volume of procedures which are being performed by different specialists in areas outside of the operating room. The delivery of sedation has evolved from the traditional narcotic, benziodiazepine, ketamine, and hypnotic agents to now include broader option of agents and routes of administration. As the choices have expanded, so also has the complexity of the challenges which face sedation care providers and, in many cases, the specialty societies which they represent. Sedation policies, procedures, and guidelines are now presented not only by specialty societies and institutions, but now also by countries themselves. 

Sedation is largely performed in areas remote from the operating room. The delivery of sedation and anesthesia in these remote areas presents risks and challenges which are unique to those of the operating room environment. In the USA, the American Society of Anesthesiologists has recognized this risk by establishing a closed claims database which collects the medicolegal outcomes of sedation or anesthesia-related events in areas outside of the operating room setting. In 2009, data from the ASA closed claims database suggests that sedation in remote locations (unfamiliar environment, inadequate anesthesia support, deficit resources, dark, small rooms, and variability of monitoring modalities) contributes to injuries and liability [73]. A review of 8496 claims concluded that sedation in remote locations is associated with a significant risk of adverse effects and a growing area of liability for the anesthesiologist [73]. 

Although specialty societies may not agree on all aspects of sedation, they all are unified by their primary interest in providing safe care. Outcome data is important in order to be able to evolve the sedation practice. To this end, the foremost challenge facing sedation care providers is the lack of universal consensus on the terminology and definition of adverse events, both minor and major. Hypoxia, oxygen desaturation, airway interventions, aspiration, and respiratory depression, for example, are all terms that are used in the literature without a universal definition. For example, some define oxygen desaturation as a drop of 10% from baseline, while others define it as an oxygen saturation less than 95% or in some cases, 90% or below. Furthermore, the duration of the desaturation often distinguishes a brief event from one which is noteworthy of being recognized as an adverse event. This duration of this desaturation is arbitrary and has not been defined or standardized. Thus, the limitations of all literature on sedation outcome is that it is based on definitions which have been established by the authors.

In order to advance the safety of pediatric sedation, through clinical studies and dedicated research, all sedation providers would benefit from having standardized descriptors of adverse events. To date, our lack of universal definitions has limited our ability to compare outcome data between different studies. Varied sedation practice and lack of consistent adverse outcome definitions have hampered our ability to evaluate the data and apply outcomes to improving sedation delivery [46, 48, 49, 74–79]. Using the same definitions to describe sedation practices, interventions, adverse events, and time intervals is an important first step to facilitate comparisons between studies and the aggregation of data from multiple studies [80–83]. The so-called “Quebec Guidelines” represented an effort to present a set of definitions which could be adopted by all sedation providers. This was a joint project between emergency medicine physicians and anesthesiologists in the United States and Canada (Consensus Panel on Sedation Research of Pediatric Emergency Research Canada (PERC) and the Pediatric Emergency Care Applied Research Network (PECARN).

 These Guidelines represented a monumental achievement-collaboration between two specialties with a consensus on terminology. Furthermore, these guidelines changed the fundamental approach to identifying and defining adverse events: they were based on the need for interventions rather than on the actual event itself [84]. This represented an important first step in establishing universally accepted terminology. 

The next step will be to reach a consensus between all specialists and their societies all over the world on the definition of adverse events. To date, these providers have operated independently, generally following the guidelines of their representing society. The Pediatric Sedation Research Consortium represents a group of institutions that voluntarily, for an enrollment fee, collect sedation data [85]. A limitation of the existing research efforts is that they are limited to those who enroll, are not large scale, and do not represent the full spectrum of specialists and sedation practice worldwide. This year, the World Society of Intravenous Anesthesia (http://www.worldsiva.org/) recognized the need to unite these specialists by establishing the International Sedation Task Force (http://www.internationalsedationtaskforce.com/). Members of this Task force share a common goal: to advance the practice of safe sedation throughout the world. 

The International Sedation Task Force represents a group of recognized sedation experts collected from around the world amongst different specialties. The members of this Task Force include sedation experts, for both adults and pediatrics: dental, hospital, emergency, gastroenterology, and intensive care medicine, as well as anesthesiology. Task force members from around the world with research and clinical expertise in sedation practice from all the major disciplines, continents and specialties are represented. The Task Force, led by Chairman and cochairman, Keira Mason, MD and Steve Green, MD, will first work to establish globally accepted definitions of adverse events which are objective, reproducible, applicable to all settings worldwide, and focused upon events which are of clinical significance. 

By establishing a common “vocabulary” to define adverse events and outcomes, sedation practice will ultimately benefit. Data will be presented in a uniform fashion which will facilitate comparison between practices globally. For example, a review of the sedation policies confirms that there are areas of disagreement: currently, the major areas of discrepancy and disagreement amongst institutions, countries, and specialty societies involves the necessary qualifications requisite of providers who deliver deep sedation and propofol. Additional discrepancies between policies involves the necessity of physiologic monitors and supplemental oxygen during sedation. To date, there is no data to support a standard which would apply across specialties. 

Establishing universal definitions will lay the foundation for someday establishing guidelines, policies and sedation boundaries: who should deliver deep sedation? Currently, many of the disagreements revolve around the debate on the whether nonanesthesiologists should deliver deep sedation or propofol. Ironically, however, the definitions of deep sedation are subjective. The sedation continuum which was established by the American Academy of Pediatrics and National Institute of Health in 1985 defines the depths of sedation using subjective criteria based on an observer's evaluation of a patient's response to tactile, verbal, and painful stimulation [54, 86]. 

The sedation continuum is an imprecise measure of sedation depth: when an emergency medicine physician or interventional radiologist provides sedation for a painful procedure, what demarcates deep sedation from general anesthesia? [87, 88] Universal definitions of adverse events will enable sedation providers to one day determine the incidence of respiratory and cardiac compromise between these levels in a step towards establishing the necessary resuscitation skills necessary for the providers of deep sedation. Furthermore, outcome data will lay the framework for reconfiguring the sedation continuum to represent an objective means of expressing depth of sedation and the associated, validated risks [89]. 

## Figures and Tables

**Table 1:** 

	Anesthesia directed	Gastroenterologist directed	Hospital medicine directed	Emergency medicine directed	Critical care directed
Sedation provider	*PHYSICIAN * Anesthesiologist *NURSE * Nurse Anesthetist	*PHYSICIAN * Pediatrician Gastroenterologist Anesthesiologist *NURSE *	*PHYSICIAN * Pediatrician Emergency Medicine Intensive care	*PHYSICIAN * Emergency Medicine	*PHYSICIAN * Intensivist *NURSE *

Training	Sedation course Pediatric Advanced Life Support (PALS)	Sedation course	Sedation course Airway management training	Educational program	No additional training
